# THE NIH PATHWAYS TO PREVENTION PROGRAM

**DOI:** 10.1093/geroni/igz038.2747

**Published:** 2019-11-08

**Authors:** Keisha L Shropshire

**Affiliations:** NIH Office of Disease Prevention, Bethesda, Maryland, United States

## Abstract

The National Institutes of Health Office of Disease Prevention (ODP) Pathways to Prevention (P2P) Program identifies research gaps, methodological and scientific weaknesses in selected scientific areas; suggests research needs; and moves the field forward through an unbiased, evidence-based assessment of a complex public health issue. P2P workshops are designed for topics that have incomplete or underdeveloped research and that have a need for a synthesis and critical assessment of the published literature. This talk will explain the purpose of this P2P workshop on use of drug therapies for osteoporotic fracture prevention within the larger context of NIH efforts to promote prevention research.

